# Stabilization of Bio-Oss^®^ particulates using photocurable hydrogel to enhance bone regeneration by regulating macrophage polarization

**DOI:** 10.3389/fbioe.2023.1183594

**Published:** 2023-06-07

**Authors:** Jiajia Wang, Xuanyu Qi, Yuqi Zhou, Guifang Wang, Yuanmeng Yang, Ting Jiang, Lei Yu, Shaoyi Wang, Wenjie Zhang

**Affiliations:** ^1^ Shanghai Key Laboratory of Stomatology, Department of Oral Surgery, Shanghai Ninth People’s Hospital, College of Stomatology, National Center for Stomatology, National Clinical Research Center for Oral Diseases, Shanghai Research Institute of Stomatology, Shanghai Jiao Tong University School of Medicine, Shanghai Jiao Tong University, Shanghai, China; ^2^ Shanghai Key Laboratory of Stomatology, Department of Prosthodontics, Shanghai Ninth People’s Hospital, College of Stomatology, National Center for Stomatology, National Clinical Research Center for Oral Diseases, Shanghai Research Institute of Stomatology, Shanghai Jiao Tong University School of Medicine, Shanghai Jiao Tong University, Shanghai, China; ^3^ School of Stomatology, Weifang Medical University, Weifang, China; ^4^ Shanghai Key Laboratory of Stomatology, Department of Preventive Dentistry, Shanghai Ninth People’s Hospital, College of Stomatology, National Center for Stomatology, National Clinical Research Center for Oral Diseases, Shanghai Research Institute of Stomatology, Shanghai Jiao Tong University School of Medicine, Shanghai Jiao Tong University, Shanghai, China; ^5^ Shanghai Key Laboratory of Stomatology, Department of Orthodontics, Shanghai Ninth People’s Hospital, College of Stomatology, National Center for Stomatology, National Clinical Research Center for Oral Diseases, Shanghai Research Institute of Stomatology, Shanghai Jiao Tong University School of Medicine, Shanghai Jiao Tong University, Shanghai, China

**Keywords:** Bio-Oss^®^, bone regeneration, hydrogel, macrophage, stabilization

## Abstract

Bone substitutes are widely used in maxillofacial and oral surgeries. However, in clinical practice, bone substitutes with various forms, including separated particulates, powders, and blocks, have exhibited poor handling properties and space maintenance characteristics, resulting in long surgery procedures and unstable volume of the newly formed bone. Movable separated particulates with high stiffness have induced local inflammatory responses that hinder bone regeneration. The present study aimed to develop a new method to enhance the stability and operability of bone substitutes commonly used in dentistry by premixing with photocurable hydrogel GelMA. The GelMA-encapsulated particulate had a strong capacity to aggregate separated particulates and firmly attach to the host bone defect after photocuring compared to particulates alone. Additionally, macrophages at the surface of the GelMA-stabilized particulates tended to present a more M2-like phenotype than those at the surface of Bio-Oss^®^, leading to more MMR^+^ multinucleated giant cell formation and the induction of blood vessel invasion and new bone formation. In conclusion, this hydrogel-coated bone substitute strategy facilitates bone regeneration with increased operability, a stable volume of osteogenic space, and a favorable osteogenic microenvironment, indicating its potential value in the field of maxillofacial and oral surgeries when bone substitutes are needed.

## 1 Introduction

The oral maxillofacial bone is prone to inflammation, trauma, tumors, and congenital disease. Defects of the craniofacial bone can occur under pathogenic conditions, such as tooth extraction, bone removal during tumor surgery, periapical bone atrophy, alveolar bone absorption, and congenital malformation of the maxillofacial region. Bone grafting is required for the replacement and regeneration of the lost bone (Janjua et al., 2022). Various bone grafting materials classified as autografts, allogenic bone, xenografts, and synthetic biomaterials have been widely investigated. Among these, autografts are considered the gold standard for bone regeneration due to associated properties of osteogenesis, osteoinduction, osteoconduction, and low immunoreactivity. Notably, however, the application of autografts is limited with respect to bone regeneration due to a low degree of acceptance of higher costs, long hospitalization times, additional surgeries, and possible morbidity at the donor site (Maiorana et al., 2019). Conversely, the disadvantages of allografts include possible disease transmission and a comparative lack of osteoinductive properties (Chaushu et al., 2010). Bio-Oss^®^ is one of the earliest and leading xenografts worldwide. It has been applied to maxillofacial and periodontal osseous defects over the past decades (Lohmann et al., 2017; Keil et al., 2021). However, due to a lack of adhesive capacity to aggregate as a firm mass, separated Bio-Oss^®^ particles are hard to achieve *in situ* immobilization under pressure from the adjacent tissue (Tejero-Trujeque, 2001). In clinical practice, complications including sinus and maxillary bone pathologies, encapsulation, and soft-tissue fenestrations had been reported to be associated with migration and/or displacement of the xenograft materials. Removal of the non-resorbed migrated particulates was the solution to the associated lesions (Rodriguez and Nowzari, 2019; Nowzari et al., 2022).

Inflammatory responses to implantation are generally believed to be associated with macrophages, the primary cell type that reaches the graft–tissue interface when biomaterial substitutes are implanted into the body (Coleman et al., 1974; Dondossola et al., 2016; Witherel et al., 2019). In the field of bone substitutes, macrophages initiate acute inflammatory reactions and switch to M1 proinflammatory macrophages (instantly to 3 days post-implantation), the prolonged M1 phase contributes to fiber encapsulation in case of impaired bone regeneration, and appropriate polarization to M2 anti-inflammation (4–7 days post-implantation) may facilitate vascularization/new bone formation (Chu et al., 2019). As a cell type, macrophages exhibit remarkable plasticity and can switch their phenotype in response to microenvironmental stimuli. Furthermore, chemical and biological stimuli, physical properties of bone grafts including stiffness, topography, confinement, and applied forces are thought to contribute to macrophage function (Zhou et al., 2021). It has been demonstrated that particulate bone grafts with well-contoured shapes reduced the proinflammatory macrophage ratio compared to sharp-edged particulates (Pujari-Palmer et al., 2016), indicating that physical injury caused by the movement of bone substitutes can induce a harmful inflammatory response. Therefore, a bone graft particulate method that incorporates separated particulates with easy handling properties and better space-making capacity that eventually reduces migration and corresponding inflammation is needed.

In addition to the inorganic component, the natural osseous tissue consists of an organic extracellular matrix (that includes collagen), which plays an important role in bone remodeling (Fonseca et al., 2014). Gelatin, a denatured collagen hydrogel (Daneault et al., 2017), has been widely used in the field of bone tissue regeneration due to its low antigenicity, low cost, and natural Arg–Gly–Asp sequence, which facilitates cell-adhesive sites (Liu et al., 2009). When grafted unsaturated bonds are modified with methacryloyl, the product is gelatin methacryloyl (GelMA), which can photo-crosslink in the presence of an initiator and a lighter of an appropriate wavelength (Dong et al., 2019), providing GelMA with spatiotemporal adhesive properties for diverse complex applications (Assmann et al., 2017; Barroso et al., 2022). Typically, the substitution ratio of the methacrylic (MA) component of GelMA is less than 5%; thus, the functional amino acid motifs of gelatin are not significantly affected, and it retains its low immunogenicity and compatibility (Liu and Chan-Park, 2010).

**SCHEME 1:**
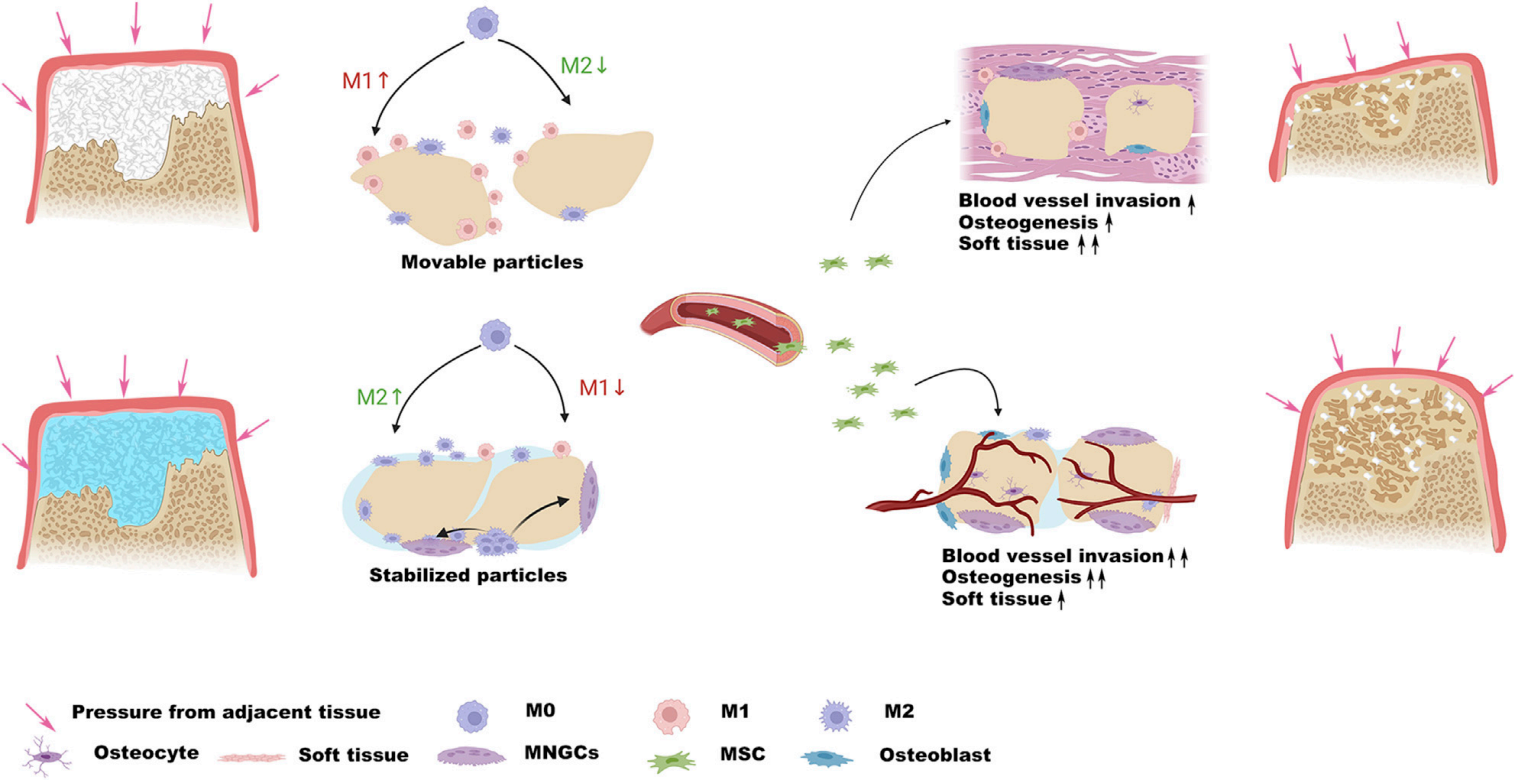
Schematic illustration demonstrates that the stabilization of the particles resists pressure from adjacent tissue and provides a favorable osteogenic microenvironment.

The present study investigated the effectiveness of photocurable GelMA as a bioadhesive for particulate-form bone graft (Bio-Oss^®^) to reduce its leakage and related inflammation. The mixture is injectable and can stably adhere to bone defects after photo-crosslinking, facilitating the surgical procedure. Subcutaneous implantation in mice demonstrated that stabilization of Bio-Oss^®^ particulates with GelMA induced a more M2-like phenotype around the implants compared with Bio-Oss^®^ alone. Moreover, a critical-sized rat calvarial defect model was used to investigate the potential of GelMA as a bioadhesive to stabilize discrete Bio-Oss^®^ particulates in the osteogenic environment driven by macrophage polarization, with the future aim of application in the field of oral and maxillofacial surgeries (Scheme 1).

## 2 Materials and methods

### 2.1 GelMA synthesis

GelMA was prepared as previously described (Yue et al., 2015). Briefly, 20 g of gelatin was dissolved in 200 mL phosphate-buffered saline (PBS) at 50°C to obtain a 10 wt% solution, followed by the addition of 16 mL methacrylic anhydride (Aladdin, Shanghai, China) and stirring for 6 h at 50°C. The reaction was terminated by adding PBS in the amount of five times the volume of gelatin solution. A 12,000–14,000 molecular weight dialysis bag was applied for 3 days to exclude unreacted small molecules, and the products were freeze-dried for 2 days and stored for further study.

### 2.2 Simulation of the application of Bio-Oss^®^ + Gel in maxillofacial surgery

To initially assess the potential application of GelMA-reinforced Bio-Oss^®^ particulates in the field of dentistry, the adherence of the compound was investigated. Bio-Oss^®^ particulates were mixed with PBS or GelMA precursor, the mixture acquired a limited adherent capacity to form a spherical mass, and then Bio-Oss^®^ particulates in the two groups were photocured using a 405-nm lighter and immersed in saline to test whether the compounds were stable. Bio-Oss^®^ was seeded into 200-µL tubes containing hydrogel or PBS to simulate alveolar ridge preservation in the context of tooth extraction, and the application of Bio-Oss^®^ in the context of existing alveolar bone loss was simulated by adding it to the bottom of the tubes. Bio-Oss^®^ adherence was tested by clamping or rinsing with saline, which are commonly used procedures during oral surgery. In addition to the aforementioned investigations, critical-sized calvarial defects in rats were utilized to further investigate the application of Bio-Oss^®^ + Gel. Bio-Oss^®^ + Gel and Bio-Oss^®^ + PBS were applied to the defects and then rinsed with saline to test adherence capacity in the two groups.

### 2.3 Scanning electron microscopy analysis

Cell morphologies in the Bio-Oss^®^ and Bio-Oss^®^ + Gel groups were observed under a scanning electron microscope. MC3T3 cells were trypsinized and resuspended in culture medium or 7% GelMA at a density of 5 × 10^6^ per 200 μL, and then 60 µL cell suspensions were seeded into 30 mg Bio-Oss^®^ particulate preparations, followed by photocuring for 20 s using a 405-nm blue lighter. Cell-loaded Bio-Oss^®^ or Bio-Oss^®^ + Gel was cultured in a medium containing 10% fetal bovine serum (Evergreen, Zhejiang, China) for 5 days. Samples were then washed with PBS and fixed with 2.5% glutaraldehyde for 4 h at 4°C, prior to dehydration with a graded ethanol series (30%, 50%, 70%, 80%, 90%, and 95%) for 15 min per step and then 100% twice for 20 min. Ethanol was exchanged with CO_2_ by using a critical point drying method (Leica EM CPD300). Lastly, the samples were coated with gold–palladium and observed using a scanning electron microscope (Phenom, Eindhoven, Holland).

### 2.4 Cell viability assays

For cell viability detection, encapsulated cells were stained using live–dead assay (Yeason, Shanghai, China) after 3 days of culture and visualized using a fluorescence microscope (Axio Scope A1, Zhejiang, China).

### 2.5 ALP staining

To investigate the effects of additional GelMA on the osteogenic differentiation of MC3T3 cells, ALP activities were tested. MC3T3 cells were trypsinized and suspended in a culture medium or 7% GelMA at a density of 5 × 10^6^ per 200 μL, and then 60 µL cell suspensions were seeded into 30 mg Bio-Oss^®^ particulate preparations, after initiation for 20 s. The cell-loaded Bio-Oss^®^ + Gel and Bio-Oss^®^ preparations were then cultured in an osteogenic medium containing 10 mM sodium β-glycerophosphate, 10 nM dexamethasone, and 50 μg/mL ascorbic acid. After 7 days of induction, the samples were washed twice with PBS, fixed in 70% ethanol for 1 h, washed twice in double-distilled water, and then incubated in BCIP/NBT (Beyotime Biotechnology, Shanghai, China) working solution at 37°C for 30 min. Images were acquired using a stereo microscope (Olympus, Tokyo, Japan).

### 2.6 RT-PCR

For Bio-Oss^®^ + cell preparation, Raw 264.7 cell suspension at a density of 5 × 10^6^ per 200 µL was directly added to the Bio-Oss^®^ particles. For Bio-Oss^®^ + Gel + cell preparation, Raw 264.7 cell suspension was suspended with 7% GelMA at a density of 5 × 10^6^ per 200 µL, and the Gel + cell compound was added to Bio-Oss^®^ particles. After initiation, the cell-loaded Bio-Oss^®^ + Gel and Bio-Oss^®^ compounds were then cultured in DMEM culture medium containing 10% fetal bovine serum for 24 h. A volume of 0.3 mg/mL GelMA lysis buffer (EFL, Suzhou, China) was added to the medium for 1 h to lysis GelMA. TRIzol reagent was used to isolate total mRNA. cDNA was prepared using Hifair^®^ V reverse transcriptase (Yeason, Shanghai, China). An ABI Prism 7500 (Bioscience) was used to perform RT-PCR. Relative mRNA expression levels were determined by normalizing to the β-actin threshold cycle and calculated using the ^△△^Ct method.

### 2.7 Immunofluorescence

Cells were seeded in Bio-Oss^®^ + Gel or Bio-Oss^®^ as previously described and induced in an osteogenic medium. On day 5, cells were gently washed twice with PBS and fixed with 4% paraformaldehyde solution for 20 min, followed by washing with PBS. Then, 0.3% (v/v) Triton X-100 was applied for 5 min to permeabilize the cell membrane. After three further washes, non-specific binding was blocked with 2% bovine serum albumin for 30 min, and then the cells were incubated with primary antibodies (anti-Runx 2, 1:200, ABclonal, Shanghai China; anti-OCN and anti-MMR, Abcam, Cambridge, UK, 1:200; and anti-iNOS, Santa, 1:200) at 4°C overnight. Cell preparations were then washed with PBS and incubated with the corresponding secondary antibody (Yeason, Shanghai, China, 1:200) for 2 h at room temperature. After three washes, the preparations were incubated in TRITC-phalloidin (Yeason, Shanghai, China) for 30 min, nuclei were counterstained with DAPI, and the cells were washed thoroughly with PBS before qualitative analysis by laser scanning confocal microscopy (Leica, Weztlar, German).

### 2.8 *In vivo* biocompatibility test

All experimental protocols were approved by the Animal Experimental Ethics Committee of The Shanghai Ninth People’s Hospital Affiliated with Shanghai Jiao Tong University. C57BL/6 mice aged 6–8 weeks underwent dorsal hair removal under anesthesia induced via inhalation of isoflurane. A 1-cm incision was made, followed by blunt dissection of the subcutaneous cell layer using blunt-tipped scissors. Bio-Oss^®^ mixed with PBS or GelMA was placed into the subcutaneous pocket. Subgroups of mice were euthanized at the terminal experimental periods 5 and 14 days after implantation. Macrophage polarization was evaluated using immunohistochemical staining for MMR and iNOS.

### 2.9 Calvarial defect model

Calvaria of male Sprague–Dawley rats aged 6–8 weeks were exposed to a trephine drill under constant irrigation, and 4-mm full-thickness craniotomy defects were created on both sides of the parietal bone, with care taken to avoid injury to the underlying dura mater. The defects were then syringed with sterile saline and filled with hydrogel-encapsulated Bio-Oss^®^ or Bio-Oss^®^ alone. All rats received drinking water containing trimethoprim-sulfamethoxazole for 3 days to prevent infection.

### 2.10 Histological and immunohistochemical analysis

Harvested samples were fixed in 10% formalin for 24 h, followed by decalcification in 10% EDTA (pH 8) for 7 days and embedding in paraffin. Samples were then cut into 4-µm-thick sections for hematoxylin and eosin (HE) and immunohistochemical staining. After deparaffinization and rehydration, sections underwent antigen retrieval with citrate antigen retrieval solution (Beyotime, Shanghai, China) at 100°C for 5 min, blocking with 5% goat serum for 1 h at room temperature, and then incubated with primary antibodies (anti-MMR, Abcam, 1:200; and anti-iNOS, Santa, 1:200) overnight at 4°C. The next day, sections were washed with PBS and incubated with secondary antibodies (Yeason, Shanghai, China) for 1 h at room temperature. Nuclei were stained with DAPI, and the sections were washed and then visualized via laser scanning confocal microscopy.

### 2.11 Statistical analysis

Unless otherwise stated, all data were analyzed using OriginPro 2018 software and expressed as mean ± standard deviation. One-way analysis of variance (ANOVA), followed by Tukey’s *post hoc* test, was used to determine the statistical significance. The significant difference was considered at *p < 0.05 and **p < 0.01.

## 3 Results and discussion

### 3.1 Bio-Oss^®^ stabilization using photocurable GelMA exhibited strong adherence and easy handling properties

**FIGURE 1:**
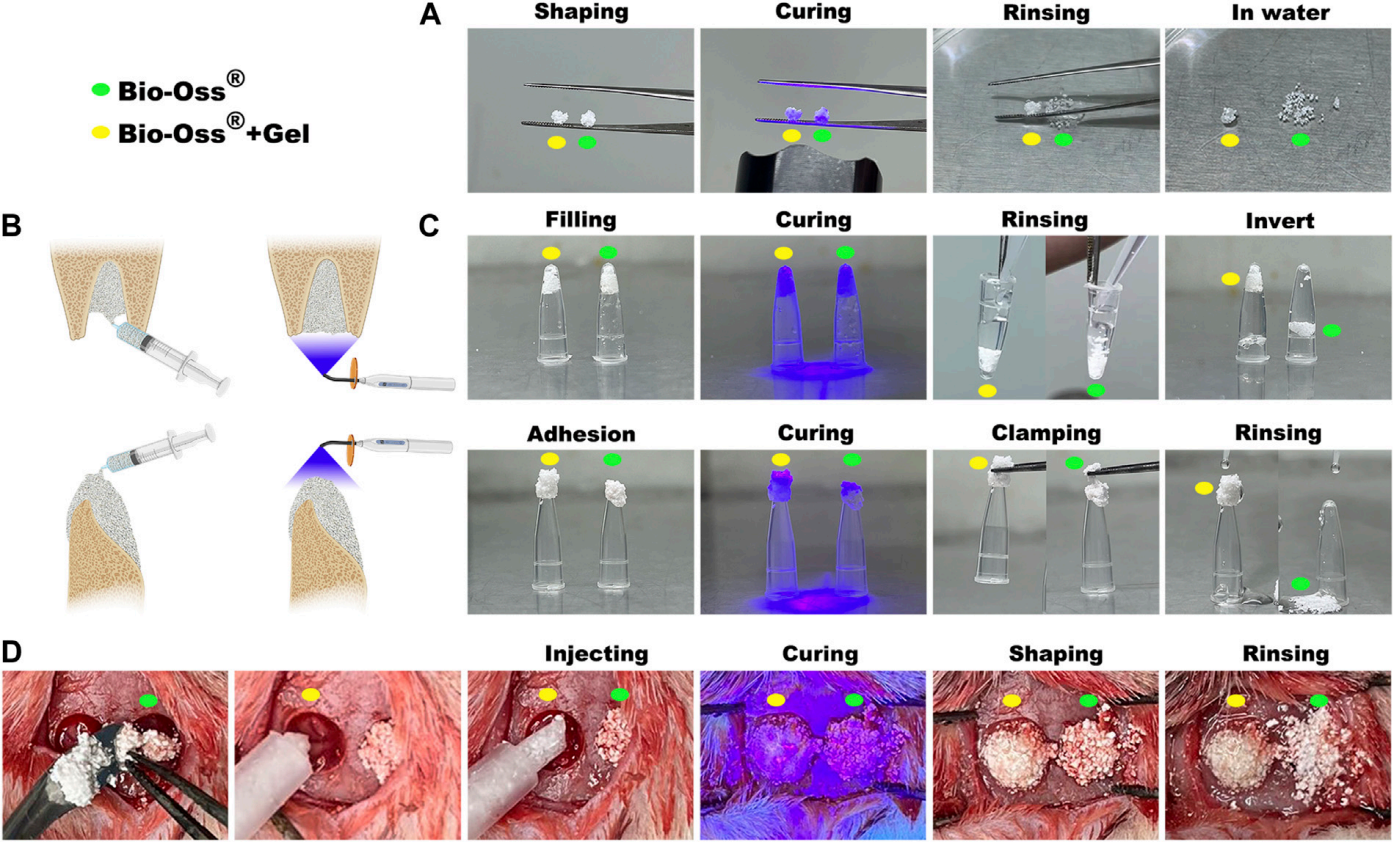
Characterization of GelMA-reinforced Bio-Oss^®^ for diverse oral applications simulated *in vitro* and *in vivo*. **(A)** Stabilization of Bio-Oss^®^ + Gel (yellow circle) and Bio-Oss^®^ (green circle) was tested by immersion in water. **(B)** Schematic diagram of some oral applications of photocurable Bio-Oss^®^ + Gel. **(C)** Simulation of Bio-Oss^®^ + Gel application in oral and maxillofacial surgery. Defects in cavity shape or non-surrounding tissue were simulated with a tube. **(D)** Critical-sized rat calvarial bone defects were filled with Bio-Oss^®^ + Gel or Bio-Oss^®^. Injectable Bio-Oss^®^ + Gel remained localized at the defect after rinsing, whereas Bio-Oss^®^ leaked out of the defect area.

The clinical applications of bone substitutes include sinus augmentation, socket/ridge preservation, and horizontal and vertical augmentation of peri-implant defects (Zhao et al., 2021). When applied to the alveolar bone, the osteogenic space should be sufficiently stable to avoid graft collapse under external tissue pressure (Urban et al., 2016). The commercially available natural particulate resorbable bone substitute Bio-Oss^®^ has demonstrated the lowest level of hydrophilicity among the naturally derived bone grafts, which compromises handling properties (Trajkovski et al., 2018). In the present study, Bio-Oss^®^ particulates gained limited adherent capacity when mixed with GelMA precursor or PBS to form a spherical mass. In the PBS group, the mass was unstable and collapsed in water, whereas, after photocuring, GelMA was successfully attached to Bio-Oss^®^ and the spherical mass was stable in water (Figure 1A). The adherence capacities of Bio-Oss^®^ + Gel and Bio-Oss^®^ in different situations that simulated maxillofacial applications were compared, including filling into the extraction socket for site preservation and application onto the absorbed alveolar ridge for vertical bone augmentation. Both Bio-Oss^®^+Gel and Bio-Oss^®^ were able to fill into the tubes, but after piping, without GelMA, Bio-Oss^®^ particulates detached from the mass. When coated with GelMA, the Bio-Oss^®^ mass remained intact after piping. Bio-Oss^®^ + Gel and Bio-Oss^®^ were then adhered onto the bottom of the tubes to simulate the application of vertical augmentation. After photocuring, Bio-Oss^®^ + Gel completely adhered to the tube and remained stable even after clamping and rinsing, whereas Bio-Oss^®^ quickly collapsed when clamped or rinsed (Figures 1A,C). In *in vivo*, Bio-Oss^®^+Gel complex was capable of being injected into the defect via a 1-mL syringe due to its flexibility. After photocuring, Bio-Oss^®^ + Gel could adhere to the defect. In the PBS group, however, Bio-Oss^®^ particulates easily spread around the defects (Figure 1D).

### 3.2 Biocompatibility of Bio-Oss^®^ alone and Bio-Oss^®^ coated with GelMA

**FIGURE 2:**
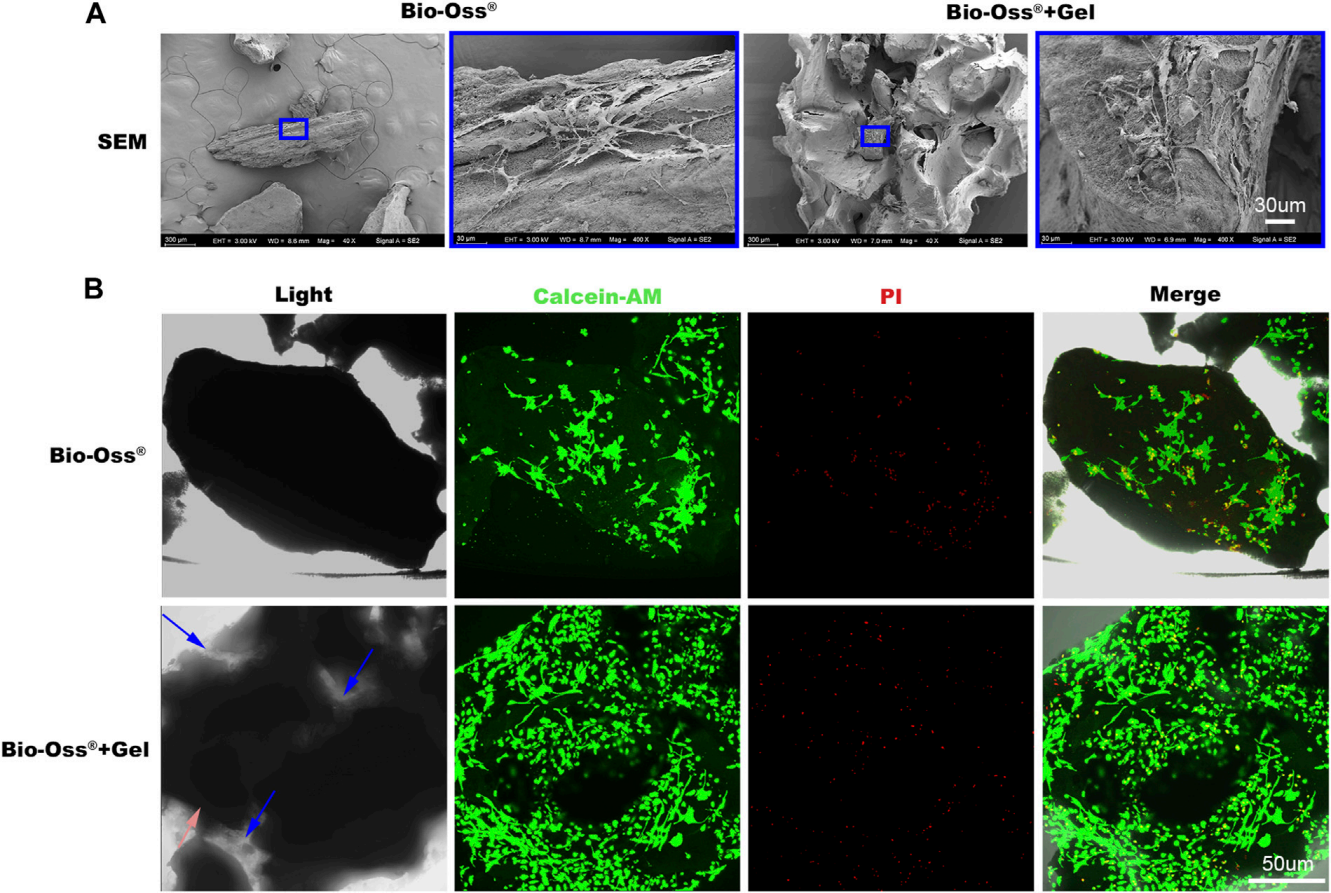
*In vitro* biocompatibility evaluation of the additional gel. **(A)** Scanning electron microscopy images of MC3T3 cells sprouting on the Bio-Oss^®^ or Bio-Oss^®^ + Gel (scale bar = 10 µm). **(B)** Images of calcein-AM PI staining of MC3T3 cells in the Bio-Oss^®^ + Gel and Bio-Oss^®^ groups (scale bar = 50 µm).

**FIGURE 3:**
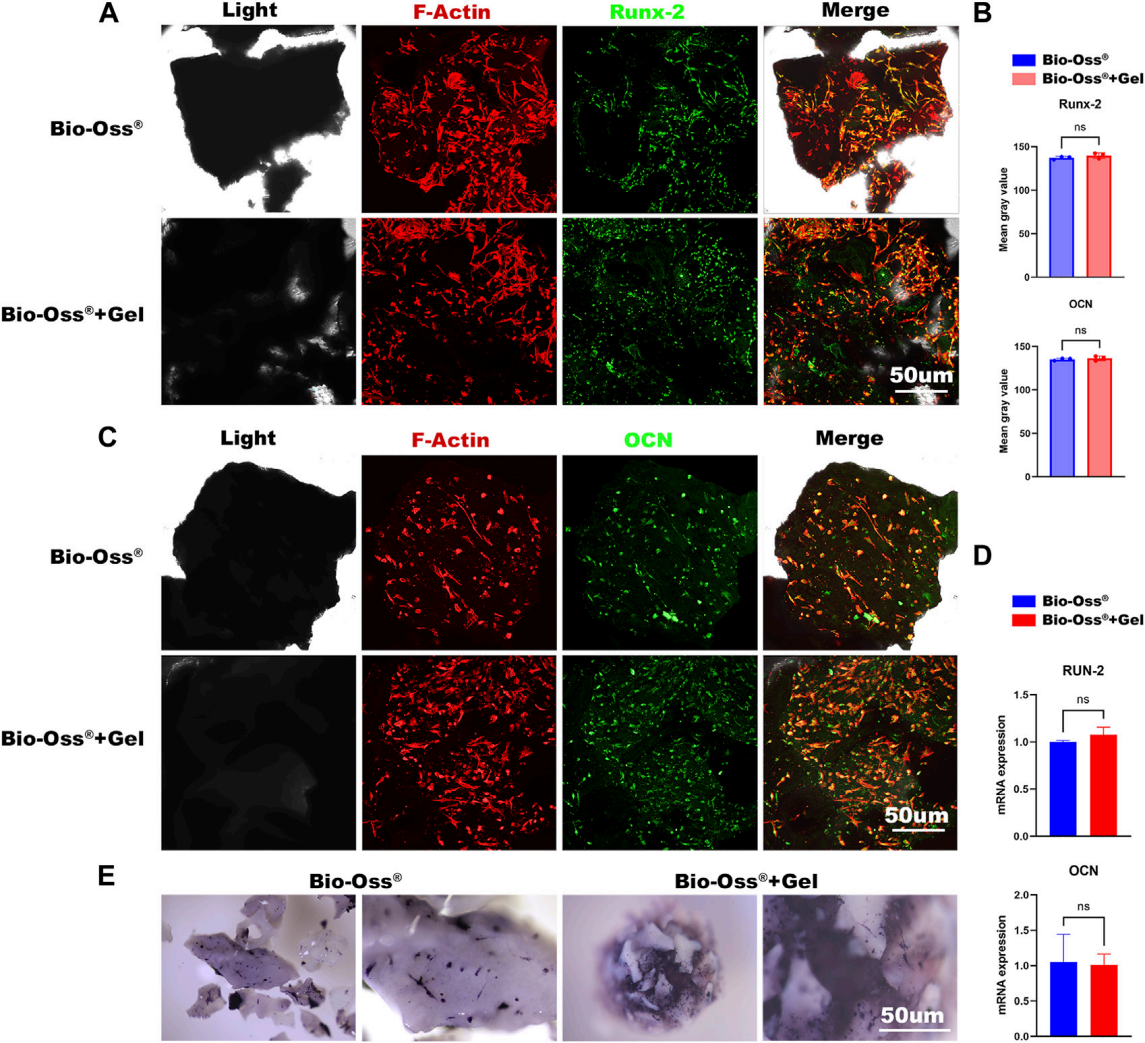
*In vitro* evaluations of the effects of additional GelMA on the osteogenic capacity of seeded MC3T3 cells. Fluorescence images and corresponding semi-quantitative analysis **(B)** of MC3T3 cells stained for Runx-2 **(A)** and OCN **(C)** in the Bio-Oss^®^ and Bio-Oss^®^ + Gel groups (scale bar = 50 µm). **(D)** Gene expression of Runx-2 and OCN detected by RT-PCR. **(E)** Stereo microscope images of ALP staining (scale bar = 30 µm).

Typically, the substitution ratio of the methacrylic (MA) of GelMA was less than 5%. At this level, the functional amino acid motifs of gelatin are not significantly affected, maintaining low immunogenicity and favorable compatibility (Liu and Chan-Park, 2010; Deng et al., 2022). Bio-Oss^®^, a xenograft harvested from different species, is processed to remove all organic components. In the present study, the effects of additional GelMA on cell viability were investigated. MC3T3 cells were resuspended in PBS or GelMA and seeded onto Bio-Oss^®^. Bio-Oss^®^ particulates aggregated together after irradiation using a 405-nm lighter for 20 s in the presence of GelMA. In the Bio-Oss^®^ group, however, separated particulates were randomly dispersed at the bottom of the medium. After culturing for 5 days, gathered particulates remained in their original shape. Cells (or cell–GelMA compound) adhered to the surface of Bio-Oss^®^ in both groups, and the living cell ratio did not differ significantly between the two groups (Figure 2). Scanning electron microscopy and cytoskeleton staining demonstrated that cells could sprout on the surface of GelMA and Bio-Oss^®^ (Figure 2; Figures 3A,B).

### 3.3 Osteogenic capacity of MC3T3 cells cultured in Bio-Oss^®^ + Gel or Bio-Oss^®^

An osteoconductive capacity to provide an environment capable of promoting mesenchymal stem cells, pre-osteoblasts, osteoblasts, and osteocytes is important concerning the function of bone grafts (Fillingham and Jacobs, 2016). To evaluate the effects of GelMA addition on the osteogenic capacity of MC3T3 cells *in vitro*, each group was osteogenically induced for 3 days or 7 days and stained for Runx-2 and OCN. Runx-2 expression is reportedly upregulated in preosteoblasts and reaches a maximal level in immature osteoblasts (Komori, 2019), whereas OCN is a later-stage osteogenic marker. In the current study, there were no significant differences in the expression of Runx-2 or OCN between Bio-Oss^®^ + Gel and Bio-Oss^®^ groups (*p* > 0.05) (Figures 3A–C). RT-PCR for the gene expression of Runx-2 and OCN also showed no significant difference between the two groups (Figure 3D). There was also no significant difference in ALP staining for osteogenesis between the two groups (Figure 3E). The results indicated that the addition of GelMA did not affect the osteogenic differentiation of seeded MC3T3 cells *in vitro*.

### 3.4 *In vitro* and *in vivo* assessments of the effects of Bio-Oss^®^ and Bio-Oss^®^ + Gel on macrophage polarization

**FIGURE 4:**
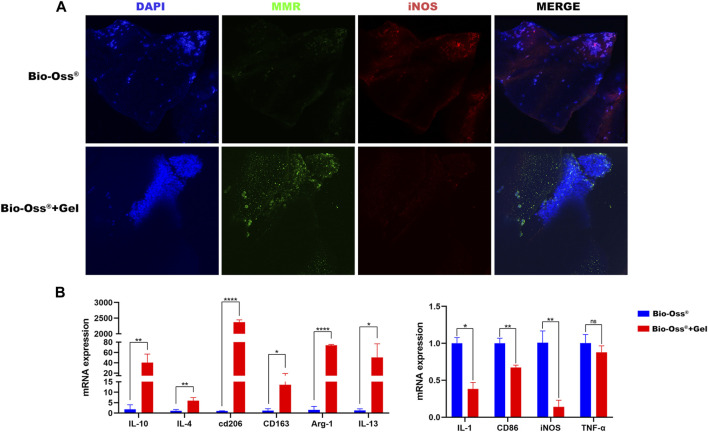
Analysis of the effects of additional GelMA on the polarization of Raw 264.7 cells. **(A)** Fluorescence images of Raw 264.7 cells stained for MMR and iNOS after 3 days of culture in the Bio-Oss^®^ and Bio-Oss^®^ + Gel groups. **(B)** Relative gene expression of M1- and M2-related markers in Raw 264.7 cells cultured on the surface of Bio-Oss^®^ or Bio-Oss^®^ + Gel for 3 days as determined by RT-qPCR. Error bars denote mean ± SD for three independent experiments. *p < 0.005, **p < 0.001, ***p < 0.0001, ****p < 0.00001, as determined by *t*-tests.

The pattern of macrophage activation dictates the outcome of implantation behavior (Coleman et al., 1974). Chemical stimuli, biological stimuli, and physical stimuli including biomaterial stiffness, topography, physical confinement, and applied forces are all thought to contribute to macrophage function (Zhou et al., 2021). The mechanosensitive capacity of macrophages has been widely studied. It is generally accepted that macrophages on a soft surface tend to exhibit reduced inflammatory traits compared to macrophages on stiffer surfaces. We hypothesized that macrophages would exhibit a more M2-like phenotype on the surface of Bio-Oss^®^ + Gel than on Bio-Oss^®^. To assess this, Raw 264.7 cells were seeded onto Bio-Oss^®^ + Gel and Bio-Oss^®^. After 3 days of culture, the protein expression levels of iNOS and MMR were visualized via immunocytofluorescence. Gene expression results at the mRNA level also confirmed a more M2-like phenotype in the Bio-Oss^®^ + Gel group than in the Bio-Oss^®^ group, based on significantly higher expression of IL-10, IL-4, MMR, CD163, Arg-1, and IL-13 and lower expression of IL-1, CD86, and iNOS (Figure 4B). MMR expression was significantly higher when Raw 264.7 cells were cultured on the surface of Bio-Oss^®^ + Gel than on Bio-Oss^®^, and iNOS expression was higher in the Bio-Oss^®^ group (Figure 4A). These results indicate more of a macrophage polarization toward M2 when cultured on the surface of Bio-Oss^®^ + Gel compared to Bio-Oss^®^.

**FIGURE 5:**
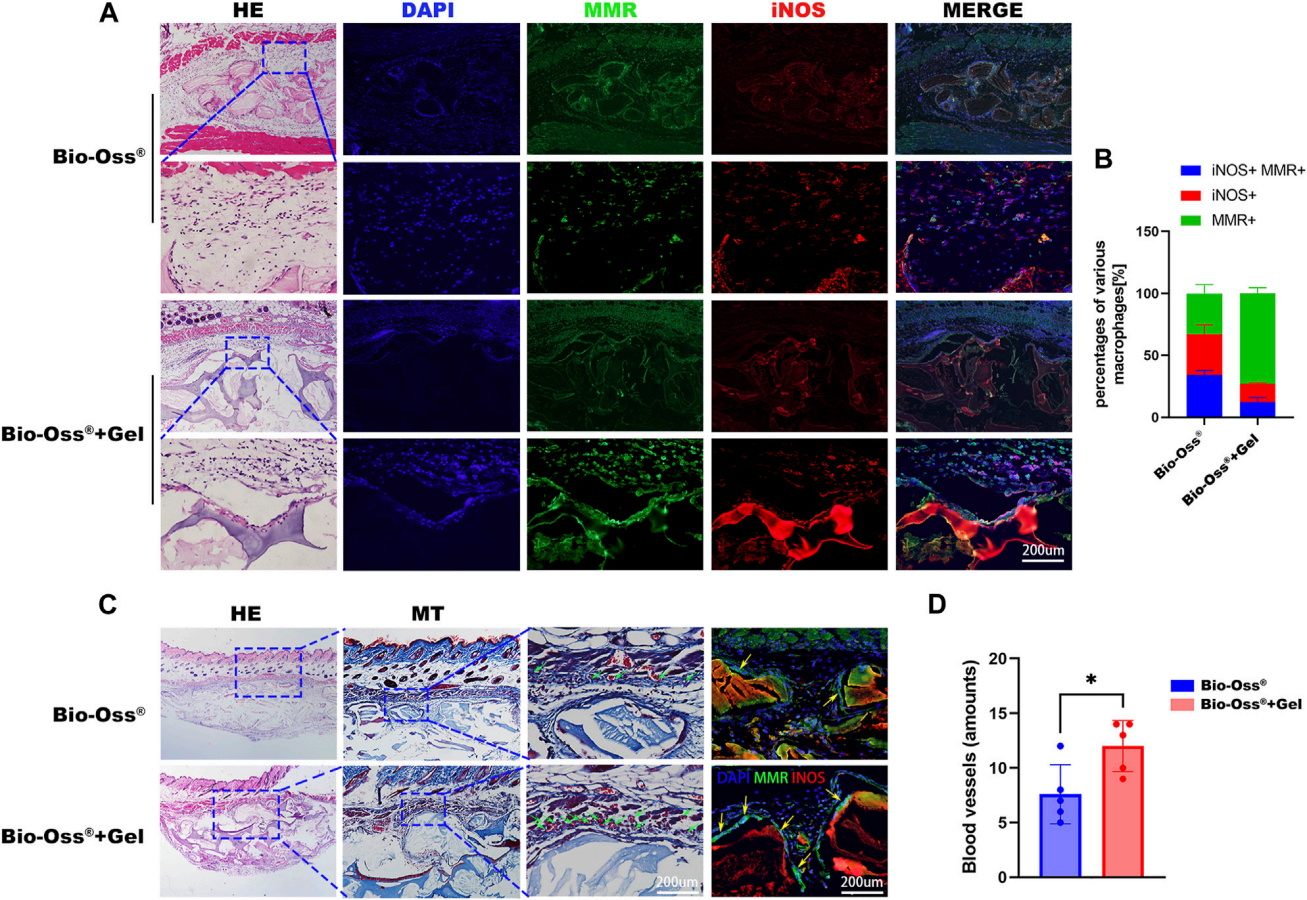
Histological analysis of subcutaneous implantation of Bio-Oss^®^ and Bio-Oss^®^ + Gel. **(A)** HE staining and immunohistofluorescence staining for MMR and iNOS 5 days after implantation (scale bars = 200 µm). **(B)** Quantification of MMR^+^iNOS^+^ cells, M1 cells (MMR^−^iNOS^+^), and M2 cells (MMR^+^iNOS^−^) (scale bars = 200 µm). **(C)** HE and Masson trichrome staining 14 days after implantation in the Bio-Oss^®^ and Bio-Oss^®^ + Gel groups. The green arrowhead indicates the blood vessel, and the yellow arrowhead indicates MNGCs (scale bars = 200 µm). **(D)** Quantification of the blood vessels around the implants. *p < 0.05, as determined by *t*-tests.

To investigate the effects of the addition of GelMA on the polarization of macrophages and angiogenesis *in vivo*, Bio-Oss^®^ and Bio-Oss^®^ encapsulated in GelMA were subcutaneously transplanted into C57BL/6 mice, a genotype known to exhibit inflammatory responses similar to humans (Vegas et al., 2016), considering that M1 macrophage markers were highly expressed at day 3, and switched to a hybrid M1/M2 phenotype with increased M2 and decreased M1 macrophages during 4–7 days post-implantation (Chu et al., 2019; Witherel et al., 2021). Macrophage polarization in response to Bio-Oss^®^ and Bio-Oss^®^ + Gel was analyzed on days 5 and 14, respectively. Macrophages surrounding the hydrogel tended to exhibit more of an M2-like phenotype, with more MMR^+^ cells 5 days after subcutaneous implantation, compared to those exposed to Bio-Oss^®^ alone (Figures 5A,B). Recent reports on surrounding multinucleated giant cells (MNGCs) in the field of bone substitute grafting tend to regard them as a key factor for the induction of vascularization, rather than an indication of a foreign body response (Barbeck et al., 2014; Miron and Bosshardt, 2018). In the present study, there were more MMR^+^ MNGCs in the Bio-Oss^®^ + Gel group than in the Bio-Oss^®^ group on day 14; moreover, blood vessels surrounding the implants were evaluated. The number of blood vessels in the tissue surrounding the Bio-Oss^®^ + Gel was significantly higher than that in the tissue surrounding Bio-Oss^®^ (*p* < 0.05) (Figure 5C). The higher density of blood vessels surrounding Bio-Oss^®^ + Gel than that around the Bio-Oss^®^ indicated higher nutrients, oxygen transplantation, and more concomitant osteoprogenitor cells, which could be beneficial for bone repair (Sivaraj and Adams, 2016).

### 3.5 GelMA-reinforced Bio-Oss^®^ promoted increased bone regeneration with more tartrate-resistant acid phosphatase-positive MNGSs in a critical-sized calvarial bone defect rat model

**FIGURE 6:**
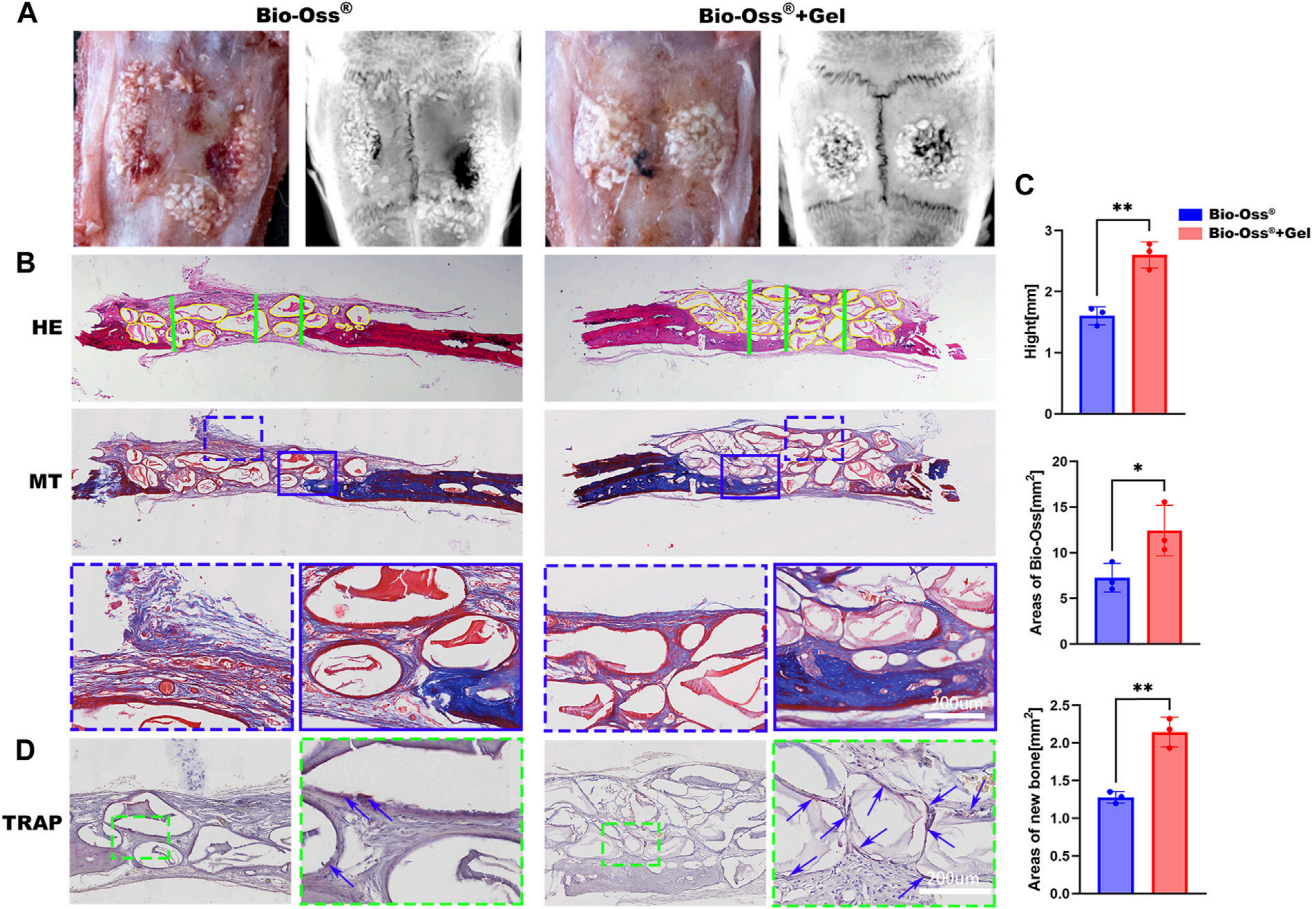
Histological analysis of the critical-sized calvarial bone defect rat model 8 weeks after implantation. **(A)** Representative gross images and X-rays from each group. **(B)** Representative images of HE and Masson trichrome staining of calvarial bone defects in the Bio-Oss^®^ and Bio-Oss^®^ + Gel groups (scale bars = 200 µm). **(C)** Quantifications of the average heights (green lines) in each group and areas of remaining Bio-Oss^®^ (yellow lines). **(D)** TRAP staining in each group. The blue arrowhead indicates MNGCs (scale bars = 200 µm). *p < 0.05, **p < 0.01, as determined by *t*-tests.

The relative capacities of Bio-Oss^®^ + Gel and Bio-Oss^®^ alone to induce new bone regeneration were evaluated using a critical-sized calvarial defect rat model. During the surgery, Bio-Oss^®^ particulates mixed with GelMA were easily passed through a 1-mL syringe and shaped with a dental explorer (Figure 1D), and the position and shape of the particulates were firmly fixed after photo-crosslinking. In the Bio-Oss^®^ alone group, particulates were motile and spread around the field of the defect. After 8 weeks post-surgery, GelMA-coated Bio-Oss^®^ particulates were intact and well-shaped in the calvarial defect area, whereas Bio-Oss^®^ particulates without GelMA had collapsed and extended outside the defect, as determined via X-ray observation and ocular manifestation (Figure 6A). HE and Masson trichrome staining confirmed that the total area of implanted Bio-Oss^®^ was larger when the Bio-Oss^®^ was encapsulated in GelMA than when it was mixed with PBS. The Bio-Oss^®^ + Gel group exhibited better maintenance of implant height and osteogenic space. There was more connective tissue in the Bio-Oss^®^ group than in the Bio-Oss^®^ + Gel group (Figures 6B,C). It has long been believed that the presence of tartrate-resistant acid phosphatase (TRAP)-positive cells around the implanted bone substitute facilitates new bone formation by modulating the osteogenic microenvironment (Huang et al., 2017; Gao et al., 2019; Qiao et al., 2021). In the present study, the Bio-Oss^®^ + Gel group exhibited more TRAP-positive cells around the implant, coinciding with more newly formed bone (Figures 6B,C). In the Bio-Oss^®^ + Gel group, the stabilized particulates exhibited a high level of operability and a strong capacity for space-making. Moreover, after stabilization with GelMA, inflammation with prolonged M1 macrophages induced by leakage and movement of the particulates was decreased. More M2-like macrophages promoted blood vessel invasion and ultimately resulted in more TRAP-positive MNGCs attached to the Bio-Oss^®^ to facilitate new bone formation.

## 4 Conclusion

In the current study, a strategy to enhance the operability and stability of bone substitutes by encapsulating them in GelMA was investigated. The injectable Bio-Oss^®^ + Gel could be adapted to various oral maxillofacial applications with reduced surgery time. This produced a favorable osteogenic microenvironment, especially concerning irregular bone defects including vertical and horizontal bone augmentation. Stabilization of the particulates by GelMA induced a more M2-like macrophage phenotype than Bio-Oss^®^ alone and increased the levels of vasculature and bone regeneration. Therefore, injectable photocurable hydrogel-coated bone substitutes have the potential for oral maxillofacial bone regeneration in clinical practice.

## Data Availability

The raw data supporting the conclusion of this article will be made available by the authors, without undue reservation.
